# Impact of Psychotherapy in Psychosis: A Retrospective Case Control Study

**DOI:** 10.3389/fpsyt.2019.00204

**Published:** 2019-04-11

**Authors:** Annbjørg Haram, Roar Fosse, Egil Jonsbu, Torstein Hole

**Affiliations:** ^1^Department of Psychiatry, Møre and Romsdal Hospital Trust, Ålesund, Norway; ^2^Division of Mental Health and Addiction, Vestre Viken Hospital Trust, Drammen, Norway; ^3^Department of Mental Health, Faculty of Medicine and Health Sciences, Norwegian University of Science and Technology, Trondheim, Norway; ^4^Clinic of Medicine and Rehabilitation, Møre and Romsdal Hospital Trust, Ålesund, Norway; ^5^Department of Circulation and Medical Imaging, Faculty of Medicine and Health Sciences, Norwegian University of Science and Technology, Trondheim, Norway

**Keywords:** global assessment of functioning, antipsychotic medication, psychotherapy, dialogue therapy, psychosis

## Abstract

**Background:** The need for psychological therapies for psychosis has become apparent since long-term antipsychotic drug treatment has a range of adverse side effects, with moderate therapeutic effects at best.

**Aims:** To investigate whether the psychotherapeutic approach, dialogue therapy (DT) is associated with improvements of symptoms and functioning beyond standard psychiatric treatment (ST) in both schizophrenia and other psychosis.

**Methods:** A retrospective case-control design, comparing 54 patients with different psychoses who received DT with 54 patients in a control group receiving ST was carried out. The groups were matched on diagnosis, age, sex, and treatment start. Outcome measures were Global assessment of functioning (GAF) scores, medications at follow up, and hospital stays after completed outpatient treatment.

**Results:** Mean time in treatment from inclusion to follow-up was 3 years and 5 months. At follow-up, GAF functioning (GAF-F) and GAF symptom (GAF-S) scores both were significantly higher in the DT group than the ST group. Effect sizes (Cohen's d) were large; 1.8 for GAF-S and 2.1 for GAF-F. At follow-up, the use of psychoactive drugs was significantly reduced despite a shorter time in psychotherapy in the DT group compared to the ST group. Days of hospitalizations after end of treatment in the study period were significantly reduced in both groups compared to the period before start of treatment.

**Conclusions:** The findings from this exploratory study are consistent with the possibility that dialogue therapy may lead to improvements in symptoms and functioning compared to standard treatment in psychosis.

## Introduction

Standard treatment (ST) for psychosis consists primarily of antipsychotics, hospitalization, social rehabilitation, and different types of supportive therapy (1–3). Antipsychotic drugs have only moderate effects on positive symptoms and no demonstrable effects on negative symptoms (4–6). Side effects are often prominent and might include a reduction in emotional expression, menstrual abnormalities, sexual dysfunction, and considerable weight gain (5). On this basis, the need for psychotherapy has become apparent (7–9).

Combinations of pharmacological and psychosocial treatments have demonstrated potential for recovery from psychosis (10, 11). A systematic review found cognitive therapy (CBT) and family interventions to improve outcome in early psychosis (12, 13). However, a Cochrane review underlined that the evidence is limited and recommended further efforts to advance the treatment of psychosis (14). In this paper, we present data on treatment effects of an original psychotherapy model, Dialogue therapy (DT).

### What Is Dialogue Therapy?

DT is an individual, dialogue oriented psychotherapy that has been developed through the first author's clinical practice and collaboration with patients diagnosed with schizophrenia and other psychoses since the 1980's (15, 16). Central sources of inspiration are humanistic traditions, language, and narrative approaches, family therapy, inter-subjectivity, and mentalization-based treatments (17–19). The treatment aims to restore health by using dialogue and collaboration to treat the illness and strengthen the patient's resources in parallel.

DT consists of three treatment phases and is provided in 1-h weekly sessions over a course that lasts between 3 months and 3 years. In the first treatment phase, the focus is on aiding the patient out of the psychosis and awakening interest in participating in a common reality. The therapist emphasizes to create an atmosphere of safety and predictability, inclusion, hope and meaning, and to invite the patient to a co-creating treatment process characterized by dialogue and collaboration. The therapist expresses empathy, compassion, authentic commitment, and sensitive curiosity toward the patient's emotions, wordless signs, and utterings. The patient is invited to tell about problems she has and is assisted in reflecting on chaotic aspects of the psychosis. The patient is complimented on progress she has made and the therapist signals a strong belief in the patient's ability for change to restore health. These issues and aspects also constitute a longitudinal fundament in the therapy that frequently is returned to in subsequent phases. The central foci in the first phase can be summarized as follows:

- Create a safe therapeutic relationship
- Communicate prospects of emotional knowledge
- Impart enthusiasm, tune in and share language
- Be genuine, show authenticity, and be responsive
- Normalize and reduce psychotic mystery and fear
- Compliment improvements, provide hope, stimulate empowerment.

Within the continued focus on establishing and maintaining a trustworthy, safe working alliance, central in the second treatment phase is to gradually include the patient in dialogue, reciprocity, and collaboration. The patient is helped to reach a greater understanding and regulation of her feelings and thoughts. The therapist is allowing for parts of the self that are dominated by the illness as well as healthy aspects of the self. By moving attention to the patient's healthy self-identity, the therapist uses emotions to stimulate the interactive process and emphasizes moments that can generate a breakthrough/splitting. This implies to help the patient in pushing symptoms aside to increase freedom and reciprocity in the dialogue and empower the patient's healthy identity. In these attempts to restore the self, the therapist personifies and visualizes symptoms to make them subjects of joint exploration, sees and compliments novel as well as previous achievements. Central foci in this phase are the following:

- Maintain a safe and predictable therapeutic relationship
- Include all the patient's narratives, life-trauma, emotional utterances, ask questions, be curious
- See the whole human being, not only the illness
- Get in between the symptoms (the illness) and the patient's healthy self-identity
- Highlight a process that helps restore a sense of self
- Externalize and help the patients label their symptoms

The third treatment phase is devoted to assist the patient back to normal life and functioning in the family and community. Independence is encouraged by increasing the scope of the dialogue and offering the patient to learn and gain insight from psychotherapeutic approaches and theories. The patient is offered assistance in searching for psychosocial explanations of symptoms in the past and present and to develop new ways of understanding. An emphasis is on strengthening the patient's belief in her own ability, resources and qualities to reestablish a meaningful life. Accordingly, various tools are provided to strengthen mental control and self-regulation to prevent relapse. This includes an emphasis on initiatives toward future work and education and other meaningful social activities. The foci of the third, final phase can be summarized as follows:

- Encourage independence
- Search for causes, free from burden
- Find explanations, evolve new histories in re-authoring lives
- Empower the patient's own qualities
- Give the patient tools from therapy and methods
- Support the journey back to normal life which includes job, educations or other activities

For a more thorough description of DT [see (15, 16)].

### A Brief Comparison of Dialogue Therapy With Other Psychotherapeutic Approaches for Psychoses

DT shares features with other psychotherapeutic approaches to psychosis but it also may have several unique features. First and foremost, DT has several meeting points with the Open Dialogue network model (20, 21). However, it differs from it with its individual psychotherapeutic orientation rather than a family and social network approach.

Shared between DT and newer psychodynamic approaches for psychosis is the emphasis on thrust, causes, history and the therapeutic alliance. The psychodynamic approaches, however, more typically view psychosis, in particular schizophrenia, as biologically based illnesses that can be managed by learning practical coping strategies (22). These models emphasize adaption and adjustment, and incorporate cognitive-behavioral multimodal theoretical orientations. Different in DT is the therapist's inclusion of the patient in an equal, exploratory collaborative context and that interpretations are part of the ongoing dialogue.

Common with DT, cognitive behavioral therapies (CBT) and metacognitive psychotherapies for psychosis typically emphasize simple and easy to understand interventions with a focus on the patient's current mental state (23–25). DT may differ from most of these approaches, however, by inviting the patient to explore experiences from the psychotic landscape as well as of trauma and history of the distant past (16, 26, 27). While traditional cognitive therapy is known for its manuals, schemes, new interpretation of settings, learning of coping strategies and strong goal-orientation (25), DT emphasizes emotions, narratives, and therapeutic alliance (17, 28). Among the approaches that may have the most in common with DT is Metacognitive Reflective Insight Therapy (MERIT) (29, 30). In MERIT, focus is on restoring the patients' integrated representations and ideas about self and others' using a range of therapeutic interventions, several of which are at least partly shared with DT, including focus on the dialogue, eliciting narrative descriptions, and stimulating to reflections about the self and about ways to understand and respond to psychological and social challenges.

## Aims

We have previously reported a larger improvement in symptoms and functioning, combined with a larger reduction in the use of psychopharmaca, in DT as compared to ordinary, standard treatment in 48 patients with a schizophrenia diagnoses (F20.0-F20.9, ICD-10) (15). In the present, extended exploratory study of DT, we present data from an additional 60 patients with a diagnosis for a psychotic disorder other than schizophrenia. Hence, we asked whether DT is associated with larger improvements in symptoms and functioning, and in larger reductions in psychopharmaca, as compared to standard psychiatric treatment in patients within the entire array of psychotic problems.

## Materials and Methods

This retrospective case-control study was conducted at the Psychiatric Outpatient Clinic (POC), Department of Psychiatry at Ålesund Hospital, Møre and Romsdal Health Trust. The hospital serves about 95,000 people from a geographical sector with both rural and urban areas. POC is a general treatment facility for all types of psychiatric conditions. Included in the study were patients enrolled to treatment at the outpatient clinic in the study period, which lasted from 1st of January 1991 to 1st of September 2008. Follow-up was defined as end of treatment or end of study period (which ever occured first). Follow up data were acquired a mean of 4 years and 1 month after treatment start. The study was approved by the National Research Ethical Committees (NEM) (2008/20) and by the Norwegian Social Science Data Services (NSD 20280). NEM and NSD approved the collection of anonymous data without patient consent. At any time point, treatment at POC is administered by an average of 25 clinicians. The majority are specialists in psychology or psychiatry, while a few are non-specialists in these disciplines, or psychiatric nurses, family therapists or clinical social workers. One person conducted DT psychotherapies (AH).

### Subjects

Eligible for inclusion in the study were patients with a diagnosis in either of the following domains (ICD-10): Schizophrenia (F20.0-9), paranoid psychosis (F22.0-9), acute polymorph psychosis (F23.0-9), schizoaffective psychosis (F25.0-9), bipolar affective disorder (F31.0-9), and severe depression with psychotic symptoms (F32.3). No exclusion criteria were used.

All patients were first considered at an intake meeting at POC, and thereafter distributed to any of the about 25 therapists working at the unit in a coincidental, unsystematic (random) manner, with no consideration of any therapist characteristics (e.g., area of specialty, experience). All patients treated with DT by the first author were included in the study, none were excluded. The control group was then matched to these patients. The intervention group received DT in addition to standard treatment (ST, see below) and consisted of all patients diagnosed with psychosis who were treated by AH (*n* = 54). The control group (*n* = 54) received ST and was selected from the total patient population with psychosis who were treated by other therapists than AH. Patients in the control group were matched to those in the intervention group on four variables in the following order of priority: 1. Diagnoses, 2. Month and year of therapy start, 3. Gender, and 4. Age. By matching the ST group on the month and year of therapy start, the two groups had the same amount of time to achieve therapeutic effects. Matching of patients was performed by an independent professional at the IT department at Ålesund Hospital, who had extensive experience from previous projects with similar mapping tasks. Characteristics of the intervention and control groups are summarized in Table 1.

**Table 1:** Baseline demographic characteristics for patients in Dialogue therapy and Standard treatment.

	**Dialogue therapy** **(*n* = 54)**	**Standard treatment** **(*n* = 54)**
Age, Mean (SD)	29.4 (10.3)	27.9 (9.6)
Female	23 (43%)	23 (43%)
Diagnosis (ICD 10)	–	–
Schizophrenia (F20.0-9)	24	24
Paranoid psychoses (F22.0-9)	10	10
Acute polymorph psychoses (F23.0-9)	5	5
Schizoaffective Psychoses (F25.0-9)	5	5
Bipolar Affective Disorder (F31.0-9)	5	5
Severe depression with psychotic symptoms	5	5

In this study, both groups received the same sort of medication therapy monitored by the same psychiatrists.

### Standard Treatment

The main focus of all treatments in ST was to stabilize the patients' mental states with antipsychotic medication, reflecting a strong biological orientation at the outpatient clinic. Usually, pharmacological treatment was accompanied by different forms of supportive or psycho-educative endeavors. The extent and concrete content of the supportive and psycho-educative approaches varied among clinicians, which included psychiatrists, psychologists, mental health nurses, and clinical social workers. However, the emphasis in all variants of treatment in ST was reality orienting dialogue and to teach the patients coping strategies to help them live as best possible with their illness. Topics such as the real life trauma and psychotic history of the patients were not addressed in any of the treatments in ST, consistent with the typical view among these clinicians that recovery was not a realistic possibility.

### Measurements

The Global Assessment of Functioning Scale (GAF) was the primary outcome measure. The secondary outcome measure was the number and dose of medications. Data also were gathered on number of admissions and days of hospitalization at psychiatric wards. All data were acquired from Electronic Patient Journals (EPJ) and paper journals by independent raters (psychiatrists).

Different psychiatrists in charge made all the diagnosis by using the International Neuropsychiatric Interview (MINI/ MINI plus), Structured Clinical Interview for DSM Disorder (SCID) and Minnesota Multiphasic Personality Inventory (MMPI-2). In addition, the appropriate diagnoses were discussed in separate diagnostic meetings that included all involved personnel. The diagnoses were retrospectively confirmed by an independent psychiatrist using DSM-III-R, DSM-IV, and ICD-9/10 criteria.

GAF is an observer-based continuous scale for the overall level of mental health/illness that ranges from 1 (most severe problems) to 100 (most healthy). Used in this study was the split-version, with separate subscales for social, occupational and school functioning (GAF-F) and mental symptom burden (GAF-S) the last week (31). The various score levels include characteristic patterns of symptom severity and difficulties of function (31, 32). First, for symptoms/ GAF-S, scores above 70 indicate general well-being and experiences of stress that represent transient, expectable reactions to psychosocial stressors. Scores from 61 to 70 indicate intermediary, moderate stress levels and symptoms of mental health problems, with scores closer to 60 reflecting e.g., fluctuating depressed mood and mild social anxiety. When moving down toward 50, typical would be occasional panic attacks and more persistent periods of depressive mood and anxieties. This would further progress with scores in the 40'ies, where it may include frequent panic attacks, recurrent suicidal ideation, and severe obsessions, worries, anxieties, and emotional dysregulation. A score of 40 usually is seen to denote the border for psychotic symptoms, including disturbed reality testing, communication and judgment, as well as hypomania, severely depressed mood, and debilitating anxiety. The domain from 40 down toward 20 reflects gradually increased severity level of a range of symptoms, including increasingly severe suicidal ideations, distorted interpersonal perceptions, delusions, paranoid ideation, dissociation, and hallucinations, with the lowest scores in this range representing highly psychotic behavioral disturbances. Scores below 20 represent imminent danger of self-destruction or death and the most urgent need of continuous help. Second, on the function subscale/ GAF-F, when scores fall down toward 60, problems start to be apparent outside the normal healthy range for social, occupational and/ or school functioning. Serious disabilities in these domains qualify for scores in the 40's, e.g., inability to comply with school demands combined with social withdrawal and recurrent aggressive behavior. Function scores below 40 represent major disability in several areas, whereas scores in the 30's reflect inability to function in almost all areas, including disability of self-care and the need to be taken care of by others.

All GAF scores were set in ordinary clinical care; however, they were decided upon as consensus ratings between at least two trained psychiatrists, a method documented to increase reliability (31). For the purpose of this study, an external, independent psychiatrist extracted the GAF scores from the patients' medical journals. A baseline score was obtained from the first evaluation documented in the patient journals after start of treatment in the study period. A second score was obtained at follow-up, defined as end of treatment or end of study period (September 1st 2008), which ever occured first.

In both treatment groups, psychopharmacological treatments were managed by different psychiatrists in charge. The prescribers did not use a shared decision making approach. We gathered information at baseline and follow up on any use of Antiepileptics (ATC code N03 A), antipsychotics (N05 A), anxiolytics (N05 B), hypnotics and sedatives (N05 C), and antidepressants (N06 A). Medication was sorted into the following subgroups: Low-dose Neuroleptics, High-dose Neuroleptics, Anxiolytics, Antidepressants, and Mood Stabilizers. All medications belonging to the same subgroup were added to derive at a summated dose for that subgroup. We also counted the total number of all psychoactive medications used.

The psychiatrist who scored the use of medications also counted the number of hospital (inpatient) admissions, the total number of days spent in hospital, and treatment duration for outpatient treatments. These data were collected from the summary of each separate admission in the medical journals. There were no evaluations involved in these extractions and registrations. All data extractions were controlled by a collaborator. Hospital admissions and days spent in hospital were calculated for two time periods. First, a baseline measure that included all life time hospital stays prior to enrolment in outpatient treatment at POC. Second, a follow up measure for the time period after end of outpatient treatment at POC in the study period. Treatment duration was defined as months in outpatient treatments at POC during the study period. Information about the duration of outpatient treatment, number of days in hospital inpatient treatment and number of hospital admissions were extracted from the patients' journals. In DT, on average, one therapeutic session was provided each week for each patient.

### Data Analysis

Differences between patients in the two treatment conditions at baseline were tested with independent sample *t*-tests for GAF and age, Chi square test for gender, and Mann-Whitney *U*-tests for medications. Mann-Whitney *U*-tests were used to test differences in the number of hospitalizations and number of days in hospital before baseline and after treatment within each study group, and differences between the two study groups were analyzed with multiple regression analyses.

To investigate impacts of treatment group upon GAF and medications we focused both on scores at follow up and on changes from baseline to follow up. First, we used independent sample *t*-tests for GAF and Mann Whitney *U*-tests for medications. For GAF, we calculated effect size using Cohen's *d*. Next, we performed more detailed analyzes with control for covariates. For GAF, we used general linear modeling, with treatment group as fixed factor and, as covariates, diagnostic group (schizophrenia, other psychoses), gender, age, number of days spent in hospital before treatment, and number of hospital stays before treatment. In these models we included as a covariate the interaction between treatment groups and diagnostic group, in order to investigate if an eventual superior effect of DT (or ST) was limited to just one of the two diagnostic groups. For medications, we used linear regression, with treatment condition, diagnostic group, gender, age, and the two noted hospitalization variables as predictors. In these analyses, we excluded duration of outpatient treatment as a covariate/ predictor since this variable was strongly correlated with treatment condition (shorter duration in DT). In multiple forward regression analyzes all factors with *p* < 0.20 were tested in the model.

All analyses were performed in SPSS v. 23.0 for Windows (SPSS Inc., Chicago, IL).

## Results

### Baseline Characteristics

At baseline, there were no significant differences between the DT and ST groups in age or gender distribution (Table 1), or in GAF scores or the use of any type of medication (left columns in Tables 2, 3). Before study baseline, the DT group had significantly higher number of hospitalizations (*p* = 0.003) and days of hospitalizations (*p* < 0.01) than the ST group. The patients in the DT group had shorter time (fewer months) in outpatient treatment during the study period compared to the ST group, median (min/max), 36 (1/132) vs. 72 (1/213) (*p* < 0.001).

**Table 2:** Changes in GAF scores over the treatment course in Dialogue therapy and Standard treatment.

	**Baseline**		**Follow-up**	
	**Dialogue therapy**	**Standard treatment**	**Dialogue therapy**	**Standard treatment**
**ALL PATIENTS (*****n*** **=** **108, 54 IN EACH TREATMENT GROUP)**				
GAF-S^*^, mean (SD)	31.2 (9.3)	32.4 (10.2)	74.9 (15.2)	47.5 (13.8)
GAF-F, mean (SD)	32.6 (9.4)	35.0 (10.5)	77.7 (15.6)	47.7 (13.0)
**SCHIZOPHRENIA (*****n*** **=** **48, 24 IN EACH TREATMENT GROUP)**				
GAF-S, mean (SD)	26.8 (9.2)	29.5 (9.3)	75.4 (15.1)	45.4 (12.8)
GAF-F, mean (SD)	28.3 (9.6)	31.6 (8.2)	77.7 (15.5)	44.7 (13.0)
**OTHER PSYCHOSES (*****n*** **=** **60, 30 IN EACH TREATMENT GROUP)**				
GAF-S, mean (SD)	34.7 (8.0)	34.7 (10.5)	74.5 (15.6)	49.1 (14.6)
GAF-F, mean (SD)	36.0 (7.9)	37.6 (11.4)	77.3 (16.0)	50.7 (13.7)

* *In both t-tests and regression analyses, at follow up, both GAF-S and GAF-F were significantly (p < 0.001) higher in patients in Dialogue Therapy compared to patient in Standard treatment. In regression analysis, these group differences were not moderated by whether patients had schizophrenia diagnoses or diagnoses for other psychosis*.

**Table 3:** Changes in medications over the treatment course in Dialogue therapy and Standard treatment.

	**Baseline**			**Follow-up**		
**Variables**	**Dialogue therapy** **(*n* = 54)**	**Standard treatment** **(*n* = 54)**	***p*-value**	**Dialogue therapy** **(*n* = 54)**	**Standard treatment** **(*n* = 54)**	***p*-value**
Low-dose Neuroleptics, Mean dose (min/max), (mg)	7.1 (0/30)	4.8 (0/24)	0.10	2.5 (0/20)	7.1 (0/50)	< 0.001
High-dose Neuroleptics, Mean dose (min/max), (mg)	95.7 (0/1,000)	46.1 (0/600)	0.14	29.5 (0/800)	185.5 (0/1,100)	< 0.001
Anxiolytics Medication Mean dose (min/max), (mg)	3.5 (0/60)	0.5 (0/30)	0.07	0.6 (0/30)	4.1 (0/45)	0.045
Antidepressants medication mean dose (min/max), (mg)	18.5 (0/190)	10.6 (0/150)	0.27	9.3 (0/225)	14.9 (0/190)	0.44
Mood stabilizing medication mean dose (min/max), (mg)	33.5 (0/900)	36.7 (0/1,650)	0.93	3.5 (0/166)	54.4 (0/900)	0.02
Number of Medications, Mean (min/max)	1.8 (0/6)	1.3 (0/4)	0.06	0.8 (0/5)	2.2 (0/6)	< 0.001

At baseline, patients with other psychoses were significantly older than patients with schizophrenia [mean age 31.0 vs. 26.0 years, *t*_(105)_ = 2.8, *p* = 0.007]. Compared to patients with schizophrenia, patients with other psychosis also had higher baseline scores on GAF-S, [mean 34.7 vs. 28.2, *t*_(106)_ = 3.6, *p* < 0.001] and GAF-F [mean 36.8 vs. 30.0, *t*_(106)_ = 3.7, *p* ≤ 0.001] (left columns in Table 2), and they used less high dose neuroleptics (*p* < 0.001) and fewer medications (*p* = 0.001) (left columns in Table 3).

### Changes in GAF-S and GAF-F

**Figure 1:**
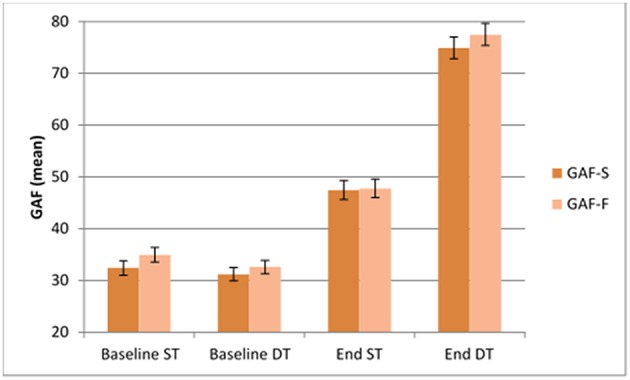
GAF scores at baseline and follow up for patients in Dialogue therapy and Standard treatment.

At follow up, the DT and ST groups differed significantly on both GAF-S [mean 74.9 (15.2) vs. 47.5 (13.8), *t*_(106)_ = 9.80, *p* < 0.001] and GAF-F [mean 77.7 (15.6) vs. 47.7 (13.0), *t*_(106)_ = 10.75, *p* < 0.001]. Both GAF-S and GAF-F also changed differently in the two treatment groups from baseline to end of therapy, in favor of the DT group, with an increase of 44.9 vs. 12.8 for GAF-S, [*t*_(106)_ = 11.12, *p* < 0.001] and 43.7 vs. 15.0 for GAF-F, [*t*_(106)_ = 9.56, *p* < 0.001], respectively (Figure 1). The effect size (Cohen's *d*) favoring DT was 1.8 for GAF-S and 2.1 for GAF-F.

At follow up there was no significant difference in GAF scores between the schizophrenia group and other psychoses.

The more detailed general linear model analysis for GAF scores at follow-up was significant for both GAF-S and GAF-F. A significant effect was seen for treatment groups upon both GAF-S (*R*^2^ = 0.47, B = 27.4, *p* < 001), and GAF-F (*R*^2^ = 0.52, B = 29.7, *p* < 001). The interaction between treatment groups and diagnostic category (schizophrenia, other psychosis) was not significant for any of the two GAF sub-dimensions, indicating a superior effect of DT over ST independent of diagnosis. No effects were seen for the covariates.

**Figure 2:**
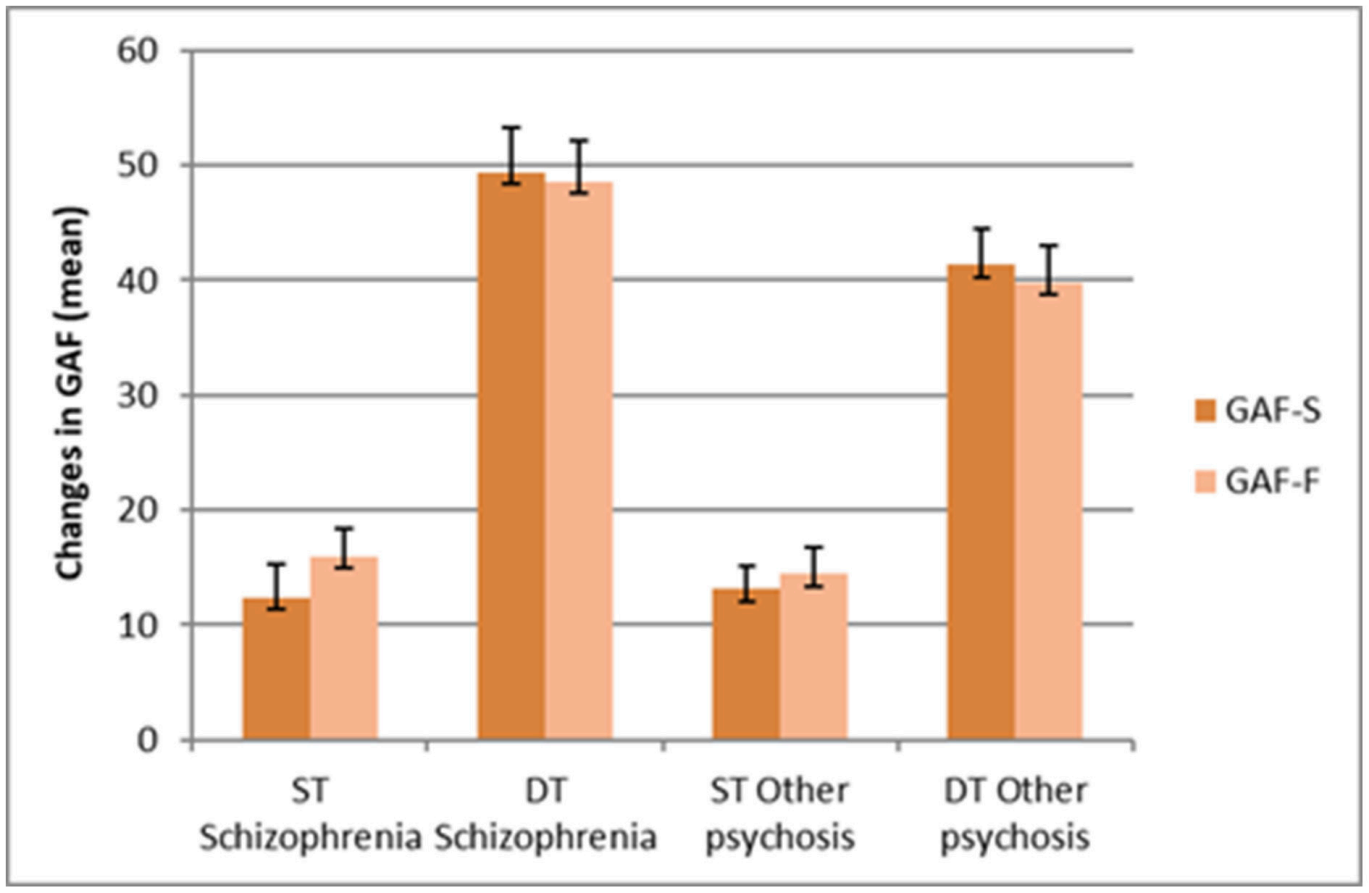
Changes in GAF scores from baseline to follow up for two diagnostic subgroups in Dialogue therapy and Standard treatment.

In each of the general linear models, with four variants of GAF as dependent variable, significant effects were seen only for treatment group; GAF-F at follow up (*R*^2^ = 0.55, *p* < 0.001), GAF-S at follow up (*R*^2^ = 0.49, *p* < 0.001), changes in GAF-S from baseline to follow up (*R*^2^ = 0.57, *p* < 0.001) and changes in GAF-F from baseline to follow up (*R*^2^ = 0.50, *p* < 0.001). Noteworthy, the interaction between treatment group and diagnostic category was not significant in any of the models, suggesting comparable effects of DT for patients with schizophrenia and for patients with other psychoses. See Table 2 for details about GAF scores at baseline and follow up and Figure 2 for changes in GAF scores from baseline to follow up, paneled by diagnostic group.

The univariate general linear model analysis for changes from baseline to follow-up also was significant for both GAF-S and GAF-F. Stronger improvements were again associated with receiving DT as compared to ST (GAF-F, *R*^2^ = 0.54, B = 32.2, *p* < 001 and GAF-S, *R*^2^ = 0.46, *B* = 28.7, *p* < 001). No interact effects were seen between treatment groups and diagnostic categories, indicating a larger improvement in GAF scores in DT as compared to ST both for patients with a schizophrenia diagnosis and patients with other diagnoses (for illustration, see Figure 2). No other covariates were significant predictors in multiple regression analyses.

### Change in the use of Medication

**Figure 3:**
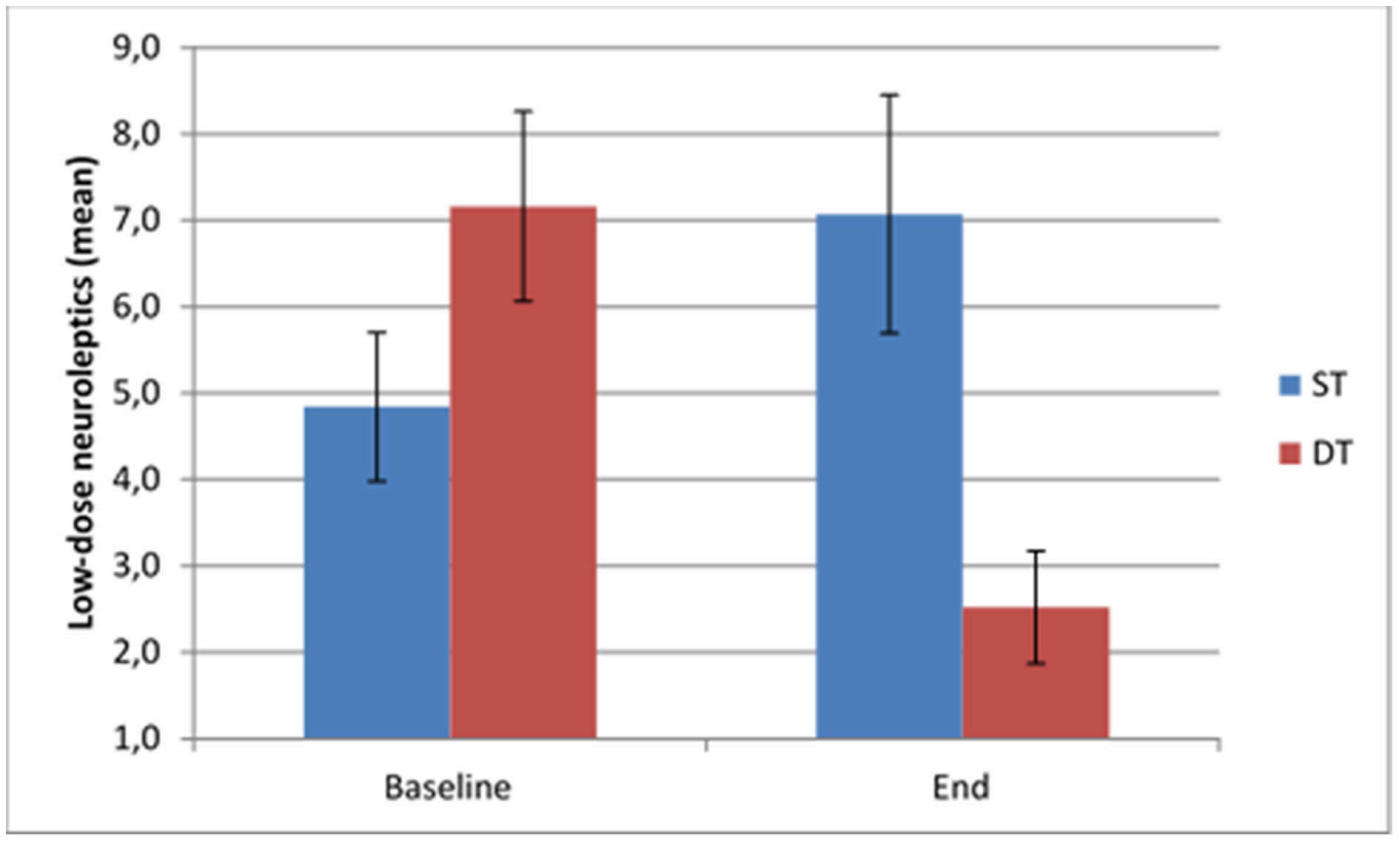
Use of low-dose neuroleptics at baseline and follow up in the two treatment groups.

**Figure 4:**
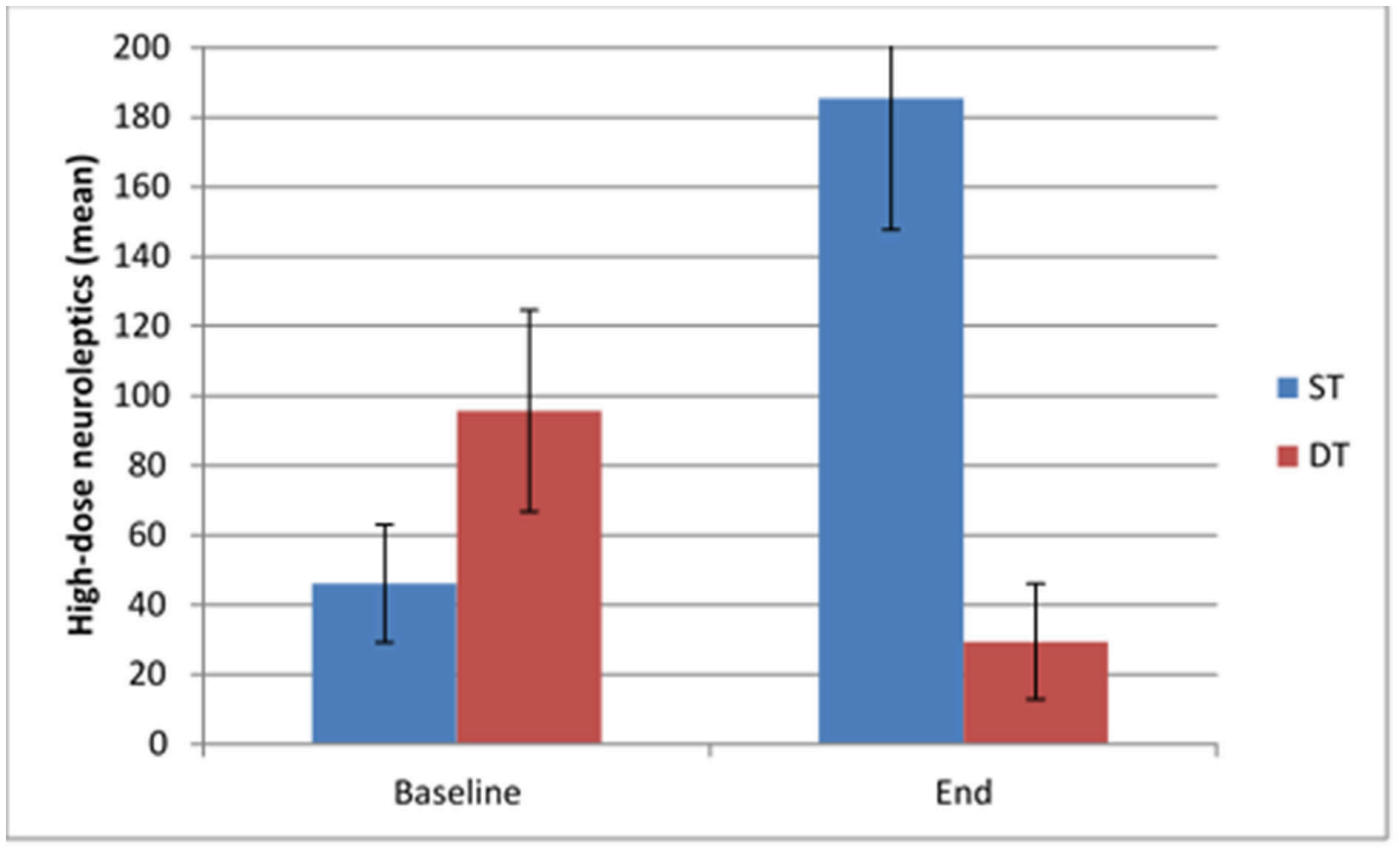
Use of high-dose neuroleptics at baseline and follow up in the two treatment groups.

At follow up, patients in the DT as compared to the ST group used less low-dose antipsychotics (*p* < 0.001; Figure 3), high-dose antipsychotic medication (*p* < 0.001; Figure 4), mood stabilizing medication (*p* = 0.02) and anxiolytics (*p* = 0.045), in addition to fewer number of drugs (*p* < 0.001). As can be seen in Table 3, medications in general increased across the treatment course in the ST group but decreased in the DT group. In statistical testing, the changes between baseline and follow up were significantly different between the treatment groups for low-dose antipsychotics (*p* = 0.001), antidepressants (*p* = 0.006), mood stabilizing medications (*p* = 0.004), and anxiolytics (*p* = 0.004), in addition to total number of drugs (*p* < 0.001).

At follow up, the univariate regression analysis for the use of low-dose neuroleptics was significant, with effects for treatment group (less use in DT) and diagnostic group (less use in “other psychoses”). In multiple regression analyses, both factors were significant (*R*^2^ = 0.11, *p* = 0.001).

The only predictor for high dose neuroleptics (*R*^2^ = 0.11, B = −156, *p* < 0.001), anxiolytics (*R*^2^ = 0.04, *B* = −3.4, *p* = 0.04) and mood stabilizing drugs (*R*^2^ = 0.05, *B* = −50.9, *p* = 0.023) in univariate and multiple regression analyses were treatment group, with less use in the DT group. The only predictor for antidepressant dose was male sex (higher dose, likely reflecting more depression in males, *R*^2^ = 0.04, *B* = 15.6, *p* = 0.031). In multiple regression analyses the total number of drugs at the end of study was predicted by treatment group and diagnostic group (*R*^2^ = 0.33, *p* < 0.001), with less medication in the DT group and in other psychoses compared to schizophrenia.

The regression analysis for changes from baseline to follow-up in the use of medication was significant for low-dose neuroleptics, antidepressants, and other medications. The only significant predictor was belonging to the DT group (lower doses) (low-dose neuroleptics, *p* = 0.003, *R*^2^ = 0.074; antidepressants, *p* = 0.009, *R*^2^ = 0.053; and other medications, *p* = 0.009, *R*^2^ = 0.053). The changes in number of drugs from baseline to follow-up was also predicted by treatment group (*R*^2^ = 0.32, *p* < 0.001), with larger reduction in the DT group.

There was no significant difference between the treatment groups in number of patients without medication before start of treatment. However, after end of therapy, there was a significant difference, with fewer patients using medication in the DT group (*p* < 0.001, Mann-Whitney *U*-test). This was true both for low-dose and high-dose neuroleptics (*p* = 0.001 Mann-Whitney *U*-test). In logistic regression analyses, therapy group (DT) and schizophrenia diagnoses were significant predictors of not using medication (*R*^2^ = 0.35, *p* < 0.001), with more patients not using medication in the DT group and in other psychoses than schizophrenia. The same was true for not using low dose and high dose neuroleptics (*p* < 0.001, *R*^2^ = 0.29, and *p* < 0.001, *R*^2^ = 0.23, respectively). The only predictor for not using mood stabilizing medication, anxiolytics, or other medications was belonging to the DT group (*p* = 0.041, *R*^2^ = 0.10, *p* = 0.050, *R*^2^ = 0.08, and *p* = 0.034, *R*^2^ = 0.07, respectively).

### Differences in Hospitalizations

During follow up, we observed more days of hospitalization in the DT group than the ST group (*p* = 0.011). In multiple regression analyses, belonging to the DT group predicted more days in hospital after end of treatment (*R*^2^ = 0.06, *p* = 0.014). However, when one extreme outlier in the DT group was removed from the analyses, no effect remained for treatment group upon hospitalization days.

## Discussion

We have previously reported larger improvements in symptoms and functioning after DT compared to ST in patients with schizophrenia diagnoses (15). In the current, extended exploratory analysis we report that in both patients with schizophrenia and in patients with diagnoses for other psychoses, larger improvements in symptoms and functioning were seen after DT than ST. Concomitant with these differences were larger reductions in the use of psychopharmaca in patients who completed DT as compared to ST, including low dose neuroleptics, antidepressants, and the number of psychoactive drugs.

Across treatment, much larger improvements in GAF scores in favor of DT were seen for both schizophrenia patients and for patients with other psychotic diagnoses. Considering the two diagnostic domains together, in the DT group, GAF symptom scores at follow up were moderate to high, representing the remaining of only mild stress symptoms and temporary and understandable reactions to psychosocial stress. Most notably, scores at the observed level indicated the general absence of psychotic symptoms and any other marked emotional and cognitive psychiatric symptoms. In contrast, in the ST group, GAF symptom scores were still low at follow up, in line with the remaining of serious symptoms in need of treatment. Regarding GAF function scores at follow up, in the DT group, they represented good functioning and only slight, if any decrease in the domains of social life, occupation, and education, with no need of assistance from the mental health system. In contrast, in the ST group, GAF function scores were still low, reflecting the continued presence of serious problems in social relations (no/ few friends) and the inability to meet normal requirements for work and studies.

The larger improvements in GAF scores in DT could not be explained with increased medical treatments since medications rather were markedly reduced in DT as compared to ST across the treatment course. Nor could it be explained with longer duration of outpatient treatment, since DT on average had shorter duration than ST. The strong improvements in symptoms and functioning in DT compared to ST, combined with the reduction in use of medication, strengthen the assumption that the effective component included psychological changes based on a psychotherapeutic process.

DT has an explicit focus on recovery from psychosis and aims both at symptom reduction through a therapeutic process oriented toward insight and self-regulation, and at helping the patient back to adequate functioning at home and in the society in general. The high GAF scores at follow up in the DT patients indicate that this goal was achieved.

Studies of treatment effects indicate that people diagnosed with schizophrenia may benefit from acquiring insight into their internal states and the external circumstances of their illness. This may help them to see causal connections and develop histories about themselves that they better can live with (16, 33), consistent with the goal of DT. We suggest that psychotherapy for schizophrenia and other psychosis should emphasize the opportunity to restore health and enable patients to develop adequate self-narratives (24, 34). It may also seek to reduce stigma and transform the language of psychopathology to a more restorative one of hope and empowerment (11, 34–37). People who experience psychosis describe stigma and attitudes from health professionals and the community related to having a schizophrenia diagnosis, as more life-limiting than the illness itself (37, 38). There is an ethical case to be made for broadening our scientific understanding of schizophrenia and other psychoses, allowing for emotions and the patient's experience of a psychosis to be more fully included in psychotherapy (3, 17, 34, 36, 38, 39).

### Strengths and Limitations

Since therapist factors may have a strong impact on outcome, a limitation is that DT involved a single therapist only; the apparent benefits of DT could alternatively reflect the particular skills and dedication of this therapist. At the same time, because only one therapist practiced DT (the founder of the model), adherence and fidelity checks have been less relevant to implement. On the other side, this has ensured a stable, comparable practice of DT for all its patients. However, the survey must be considered preliminary and exploratory, and controlled prospective studies that include more therapists providing DT are needed. Strengths include that all patients who received DT and fulfilled criteria for psychosis, were included in the study, and that the ST group was matched on several criteria to the DT group. However, the likely varied approaches in ST makes it difficult to know exactly what DT was compared to. A further limitation is that although GAF scores were set in consensus by at least two trained professionals, this was done in ordinary clinical care, with no independent scores set by researchers. Other weaknesses are that patients were not allocated to treatment groups using conventional randomization methods; the small size of the sample investigated; the limited range of outcome measures; and the dependence of the outcome measures on information in the clinical notes. Moreover, even if strengths include that all patients who received DT and fulfilled criteria for psychosis were included in this study, the limited range of outcome measures does not allow deepening the complexity of the sample, which includes the entire psychosis spectrum. We had no measure of the proportion of patients in ST who received psychoeducation and medication vs. medication only. Thus, suboptimal aspects of ST for some patients may have contributed to this group's worse outcome compared to DT.

## Conclusions

In this preliminary and exploratory study, compared to standard treatment, the psychotherapeutic approach Dialogue therapy was associated with improved functioning and reduced levels of general symptoms at follow up in both patients with schizophrenia and patients with other psychosis. The differences were seen in spite of reduced use of medication and shorter duration of therapy in DT. These promising findings for DT warrant subsequent controlled studies that include larger patient groups and more therapists in order to conclude about effects.

## Ethics Statement

The project was approved by the National Research Ethical Committees (NEM) (2008/20) and by the Norwegian Social Science Data Services (NSD 20280). NEM and NSD approved the collection of anonymous data without patient consent.

## Author Contributions

AH, TH, and RF have made substantial contributions to conception, design, analysis, interpretation of data, and agreed to be accountable for all aspects of the work in ensuring that questions related to the accuracy or integrity of any part of the work are appropriately investigated and resolved. AH, TH, EJ, and RF have been involved in interpreting the data and drafting the manuscript or revising it critically for important intellectual content. All authors read and approved the final version of the manuscript.

### Conflict of Interest Statement

AH has developed the new psychotherapeutic approach and published a book about the method. The remaining authors declare that the research was conducted in the absence of any commercial or financial relationships that could be construed as a potential conflict of interest.
